# Bioinformatic profiling identifies the glutaminase to be a potential novel cuproptosis-related biomarker for glioma

**DOI:** 10.3389/fcell.2022.982439

**Published:** 2022-09-09

**Authors:** Zhen Ouyang, Hanyi Zhang, Wenrui Lin, Juan Su, Xianggui Wang

**Affiliations:** ^1^ Department of Dermatology, Hunan Key Laboratory of Skin Cancer and Psoriasis, Hunan Engineering Research Center of Skin Health and Disease, Xiangya Clinical Research Center for Cancer Immunotherapy, Xiangya Hospital, Central South University, Changsha, China; ^2^ National Engineering Research Center of Personalized Diagnostic and Therapeutic Technology, Changsha, China; ^3^ Eye Center of Xiangya Hospital, Central South University, Changsha, China; ^4^ Hunan Key Laboratory of Ophthalmology, Xiangya Hospital, Central South University, Changsha, China

**Keywords:** glutaminase, cuproptosis, long non-coding RNAs, neuroinflammation, nonprogrammed cell death, immunity

## Abstract

Glioma is the most common tumour of the central nervous system, with a poor prognosis and an increasing trend of incidence in recent years; it is also beginning to affect younger age groups more. Added to this, cuproptosis is a new form of cell death. Indeed, when a certain amount of copper accumulates in a cell, it affects specific mitochondrial metabolic enzymes in that cell and leads to cell death–a phenomenon known as cuproptosis. In this study, we applied bioinformatics analysis, and, according to the results of the study analysis and Gene Ontology (GO), as well as the Kyoto Encyclopedia of Genes and Genomes KyotoEncyclopediaofGenesandGenomes, the glutaminase (GLS) genes affect the prognosis and tumour mutation of glioma patients through cuproptosis. Interestingly, however, GLS is not involved in the immune escape of glioma. Glutaminase genes are a class of glucose metabolism-related genes that are involved in the tricarboxylic acid cycle of cells. At the same time, the expression of the glutaminase gene was positively correlated with the degree of immune cell infiltration and the expression of various immune cell markers, and thus affected the prognosis of glioma patients. Therefore, we believe that the cuproptosis-related glutaminase gene can be an important factor in determining the prognosis of glioma patients.

## Introduction

Central nervous system (CNS) malignancies are the cancers with the worst prognosis. It is estimated that any brain tumour-related cancer results in the highest number of years of life lost (averaging approximately 20 years) (Wen et al., 2020). Glioma is graded, according to histological and clinical criteria, as grades I to IV. Grade II and III glioma is designated as low-grade glioma (LGG). Although LGG accounts for a minority of glioma, it is the leading cause of death in young adults (An et al., 2020) (Ostrom et al., 2016). Glioma encompasses the most common primary central nervous system tumours, with glioblastoma being the most malignant glioma (WHO grade IV), and the annual incidence of glioblastoma in the United States is estimated to be 6.6 per 100,000 people (Ostrom et al., 2014) (Gusyatiner and Hegi, 2018). Glioblastoma (GBM) is the most invasive and common form of brain cancer in adults, and there is a lack of highly effective treatment options (Pang et al., 2019) (Lee et al., 2018). Currently, the common treatment for glioma is surgery, but patient prognosis varies widely, with a median survival of less than 2 years at the current maximum safe resection criteria (Stupp et al., 2009) (Reifenberger et al., 2017). For glioma with higher malignancy, we urgently need relevant targeted therapeutic support to improve the prognostic survival time of patients. However, the efficacy of gene-targeted therapy for glioma patients is not ideal at present (Nicholson and Fine, 2021) (Chatwin et al., 2021) (Poff et al., 2019). Therefore, there is an urgent need to explore new prognostic predictors and therapeutic targets for glioma. In this study, we try to explore the relationship between glioma and cuproptosis.

Cell death is an important step in the development of an organism and is closely related to the immunity of the organism. Among these, cell death induced by copper ion carriers (cuproptosis) is a new pathway of cell death, clearly distinguished from the traditional ways of death (apoptosis, ferroptosis, necrosis). The acquisition, distribution, and elimination of the metallic trace element copper are vital for life (Abbott et al., 2021) (Wiebelhaus et al., 2021). Copper is an essential trace element required for many key enzymes, including cytochrome c oxidase, which generates the electron transport pathway adenosine 5′-triphosphate (ATP) in mitochondria (Kim et al., 2008) (Gaetke et al., 2014) (Guthrie et al., 2020). Paradoxically, copper is intrinsically toxic, in part because of its ability to generate hydroxyl radicals in biological systems. Excess copper accumulation triggers the destruction of iron-sulfur cofactors and stimulates the production of damaging reactive oxygen species via copper-driven Fenton reactions. Known mechanisms and enzymatic targets of copper cannot fully explain the cellular response to copper toxicity (Cobine and Brady, 2022). When intracellular copper accumulates to a certain level, the destruction of specific mitochondrial metabolic enzymes results, triggering an unusual mechanism of cell death referred to as cuproptosis (Kahlson and Dixon, 2022) (Tsvetkov et al., 2022) (Tsvetkov et al., 2019). The concept of cuproptosis provides new ideas for the mechanism and the exploration of the treatment of glioma and even all tumours.

The glutaminase (referred to as GLS hereafter) product is responsible for participating in the cellular tricarboxylic acid (TCA) cycle along with glutamine-derived a-ketoglutarate, thereby supporting cellular energy production and meeting biosynthetic requirements, thus promoting tumour cell growth and progression (Mafra and Dias, 2019) (Cluntun et al., 2017). The dependence on glutamine has long been recognised as a hallmark of cancer cell metabolism and plays an important role in tumour development (Binkley et al., 2020) (Schultze and Aschenbrenner, 2021), during which GLS is frequently overexpressed in many malignancies and acts as an oncogene to support cancer growth (Gao et al., 2009) (Wang et al., 2010). GLS has been shown to be regulated transcriptionally, post-transcriptionally and post-translationally through various mechanisms, such as long non-coding RNAs (lncRNAs); lncRNAs constitute a non-coding RNA greater than 200 nucleotides in length (Wang et al., 2021) (Sanchez Calle et al., 2018). The specific mechanism of action between cuproptosis-related lncRNAs and GLS remains to be explored in further studies (Deng et al., 2019). In this work, we try to investigate and analyse the relationship between GLS genes and cuproptosis-related lncRNAs from the perspective of cuproptosis, and to further explore this relationship’s role in the progression of glioma.

## Methods and materials

### Data collection

Relevant cohort data was collected from The Cancer Genome Atlas (TCGA) database (https://portal.gdc.cancer.gov), with a total of 706 cases of LGG and GBM RNA-seq data (Transcriptome profiling, Gene Expression Quantification), a total of 1,114 cases of clinical group data (clinical, Clinical Suplement), and a total of 991 cases of mutation group data (simple nucleotide variation, Masked Somatic Mutation). Immune escape-related data was obtained from the Tumor Immune Dysfunction and Exclusion (TIDE) database (http://tide.dfci.harvard.edu/login/).

### Differential gene analysis and construction

RNAseq expression profiles of gliomas after normalization were compared using the DESeq2 package. To normalize gene expression in TCGA, transcripts per kilobase per million fragments (FPKM) were converted to transcripts per million (TPM) using the corresponding formula. TPM is first corrected count value according to gene length, i.e., the number of corrected reads is obtained by dividing count value by gene length (in millions), adding all corrected count values to obtain corrected total counts value, and dividing corrected count value by corrected total counts value to obtain TPM value.

### Prognostic survival model and prognostic analysis

The caret package and glmnet package in R (R for Mac OS X GUI, version 4.1.0) were used to perform lasso regression analysis, one-way and multi-way cox analysis and construct a prognostic cox model on the grouped cohort data (From a random grouping). Next, we analyzed and compared the prognosis and survival time of glioma patients in high and low risk groups by using survival package and survminer package (version 4.1.0), and concluded that *p* value less than 0.001 was significant.

### Gene ontology term and Kyoto Encyclopedia of Genes and Genomes pathway enrichment analysis

To explore the potential biological processes and molecular functions of GLS genes in the enrichment of genes, gene ontology (GO) functional analysis and Kyoto Encyclopedia of Genes and Genomes (KEGG) pathway enrichment analysis were performed using the CLUSTER” ProfilerR package. The data were visualized and analyzed with circlize, ggplot2, and ggpubrR packages. An adjusted *p* < 0.05 was considered to indicate statistically significant differences in the graphs. In total, the GO database has three major categories, BiologicalProcess (BP), CellularComponent (CC), and MolecularFunction (MF), each describing KEGG (KyotoEncyclopediaofGenesandGenomes) is a database for systematic analysis of gene function and genomic information, which helps researchers to study gene and expression information as a whole network. Another task of KEGG is a process of linking a series of genes in a genome with a network of intracellular molecular interactions, such as a pathway or a complex, by them to reveal a higher level of biological function.

### Statistical analysis

Statistical analysis was performed with R software (R for Mac OS X GUI, version 4.1.0). Shapiro-Wilk test is used to estimate the normal distribution of continuous variables, and Bartlett test is used to estimate the homogeneity of variance. According to the data homogeneity of variance and normal distribution, independent sample *t*-test or Wilcoxon symbolic rank test was used. Survival analysis was performed by LOG-rank test. The t‐test with significance at *p* < 0.05. * means *p* < 0.05, ** means *p* < 0.01 and *** means *p* < 0.001.

## Result

### Construction of a prognostic model for patients with glioma

**FIGURE 1:**
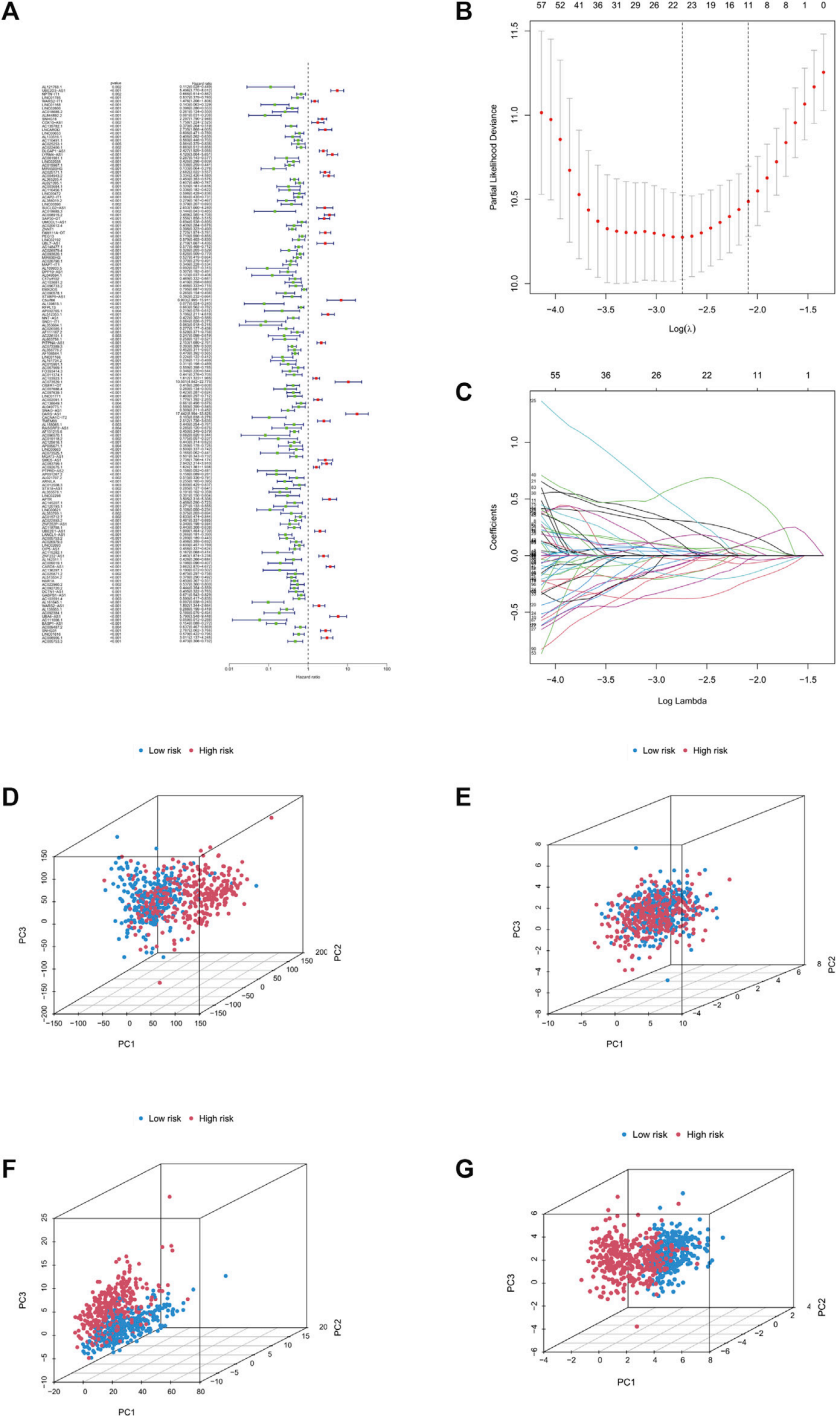
Construction of prognostic models. **(A)** Forest plot, green for low risk lncRNAs, red for high risk lncRNAs. **(B)** Lasso regression graph. **(C)** Cross-validated graph. **(D)** Graph of all genes. **(E)** Graph of copper death-associated genes. **(F)** Copper death-associated lncRNA model. **(G)** Model lncRNA model. Abbreviation: lnc RNAs, Long non-coding RNAs.

We analysed and integrated the relationship between clinical data and lncRNA expression of cuproptosis-related genes in 1,114 LGG and GBM patients, using one-way and multi-way cox analysis, as well as lasso regression analysis. The filtering criterion for significance of the one-way cox method was set at 0.05, followed by the construction of a cox model regarding the prognosis of LGG and GBM patients, according to which patient risk factor correlation was scored, and patients were divided into high- and low-risk groups according to the median value of the scores. Furthermore, the difference in survival between the high- and low-risk groups was compared to obtain the *p*-value of the difference, and we set *p* < 0.001 as the criterion for inclusion in the model. Through this analysis, we were able to determine that lncRNAs such as DARS-AS1, UBE1D3-AS1, and LYRM4-AS1 were significant high-risk lncRNAs (Figure 1A). In total, 11 of the most significant lncRNAs (Figures 1B,C) were screened based on the results of lasso regression. Principal component analysis was then performed to distinguish between high- and low-risk group patients (Figures 1D–G).

### Analysis of risk differences in patients with glioma

**FIGURE 2:**
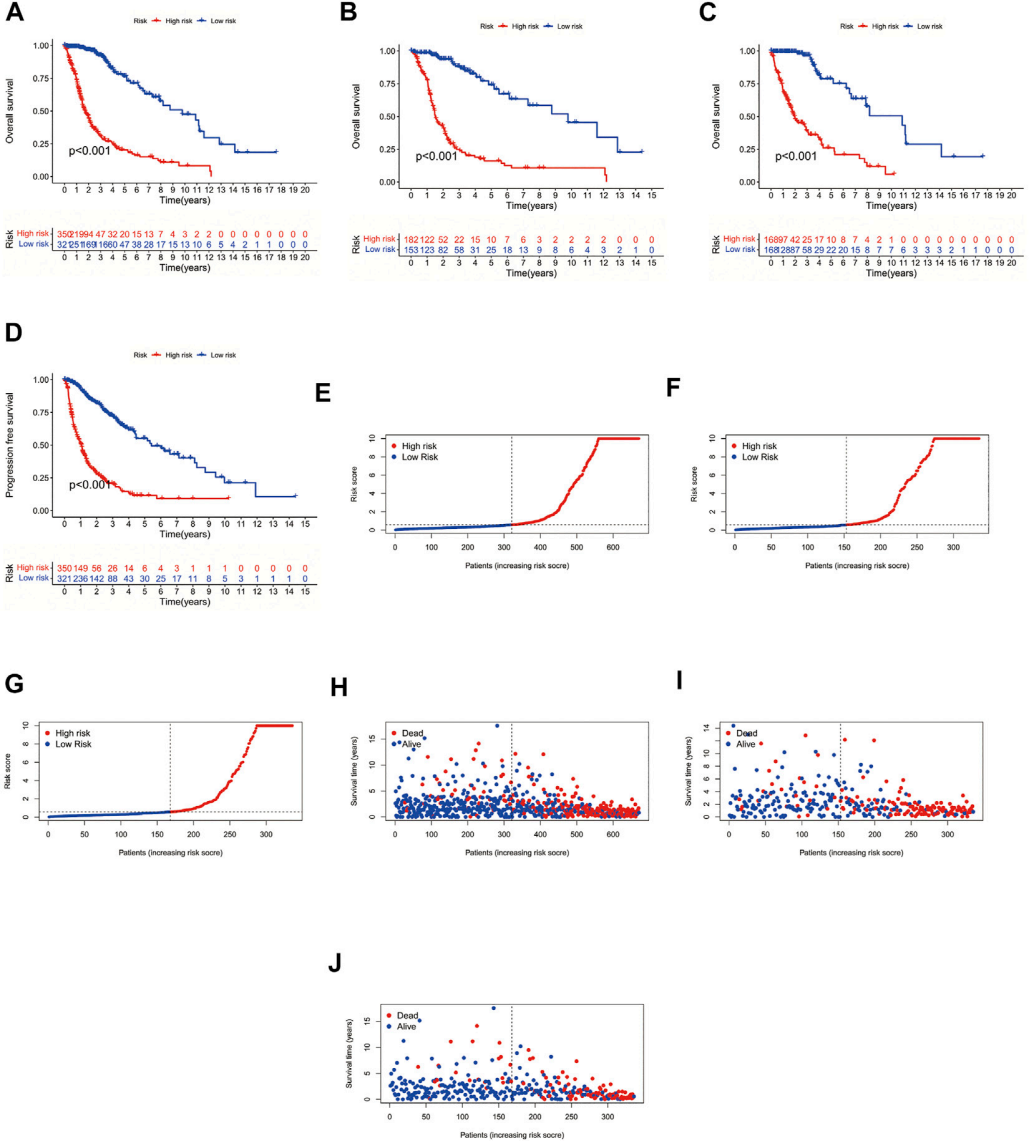
Survival curves. **(A)** Overall patient survival curves. **(B)** Survival curves for patients in the Test group. **(C)** Survival curves for patients in the Train group. **(D)** Progression-free survival curves. **(E)** Overall patient risk curves. **(F)** Risk curves for patients in the Test group. **(G)** Risk curves for patients in the Train group. **(H)** Overall patient survival status graph. **(I)** Survival status graph for patients in the Test group. **(J)** Survival status graph for patients in the Train group.

Using the survival, survminer package in R, we compared the survival differences between patients in the high- and low-risk groups, and obtained a significant *p*-value (*p* < 0.001). We found significant differences in the prognosis of patients in the high- and low-risk groups amongst the overall patients, the patients in the train group, and the patients in the test group, with patients in the low-risk group having a better prognosis than those in the high-risk group (Figures 2A–C). Subsequently, we performed a prognostic analysis of patients with progression-free survival (PFS), comparing the difference in PFS between the high- and low-risk groups; we obtained a significant *p*-value (*p* < 0.001), indicating that there was a significant difference in the prognosis of patients with PFS between the high- and low-risk groups, and that patients in the low-risk group had a better prognosis than those in the high-risk group (Figure 2D). Subsequently, we performed risk analysis and assessment of patients, finding that patients in the high-risk group had significantly different risk scores and survival times than did patients in the low-risk group, whilst patients in the low-risk group had lower risk scores than did those in the high-risk group, and patients in the low-risk group had longer survival times than did those in the high-risk group (Figures 2E–J).

### Association between GLS and cuproptosis-associated lncRNAs

**FIGURE 3:**
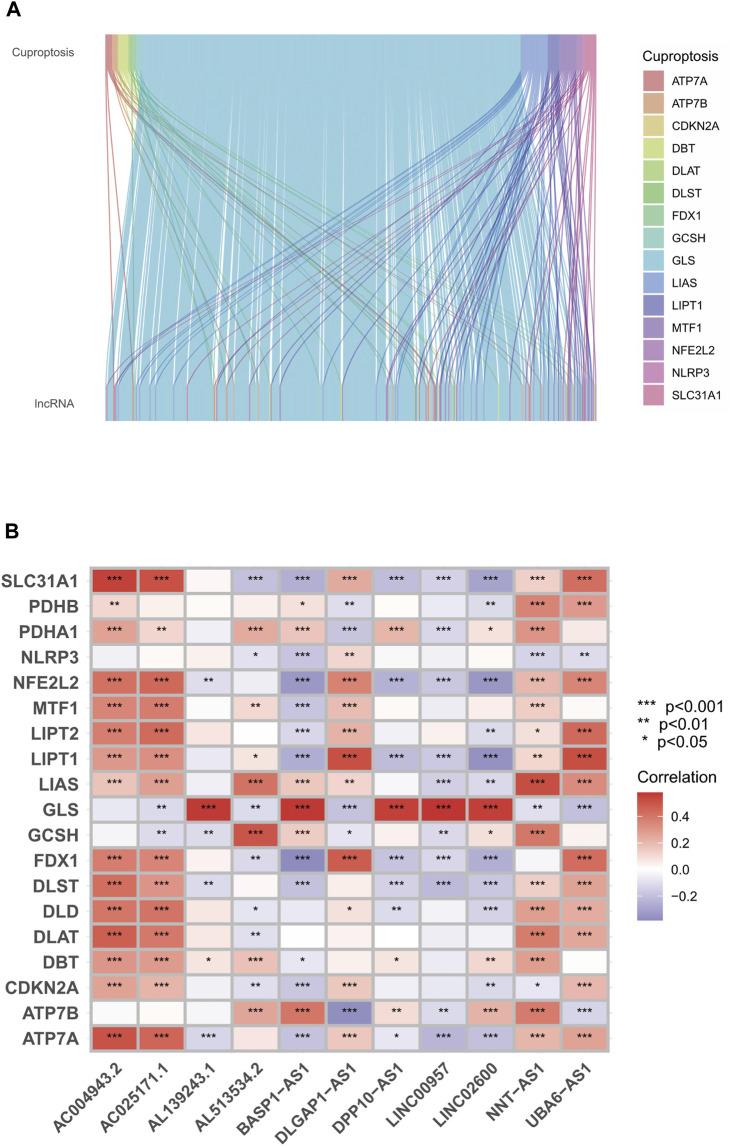
Relationship between GLS genes and copper death-related lncRNAs. **(A)** Sankey diagram of co-expression relationship between cuproptosis-related genes and lncRNAs. **(B)** Heat map of copper death-related genes and lncRNA correlation. ***p < 0.001, **p < 0.01, *p < 0.05. Abbreviation: GLS, Glutaminase.

After correlation tests and differential analysis with cuproptosis-related genes, we extracted cuproptosis-related lncRNAs from the TCGA cohort. Subsequently, continuing with the correlation tests, we derived co-expression relationships between cuproptosis-related genes and lncRNAs in LGG and GBM (Figure 3A). As shown by the results, the co-expression of GLS, LIPT1, MTF1 genes and glioma cuproptosis-related lncRNAs was the most significant. Amongst them, GLS was the most prominent. According to the established prognosis-related cox model and correlation test, we obtained the co-expression relationship between cuproptosis-related genes and lncRNAs, amongst which the correlation of GLS was the most significant (Figure 3B). From the previous prognosis and risk analysis, we learned that GLS was significantly correlated with the prognosis and risk of glioma patients.

### GLS is involved in cuproptosis in glioma cells

**FIGURE 4:**
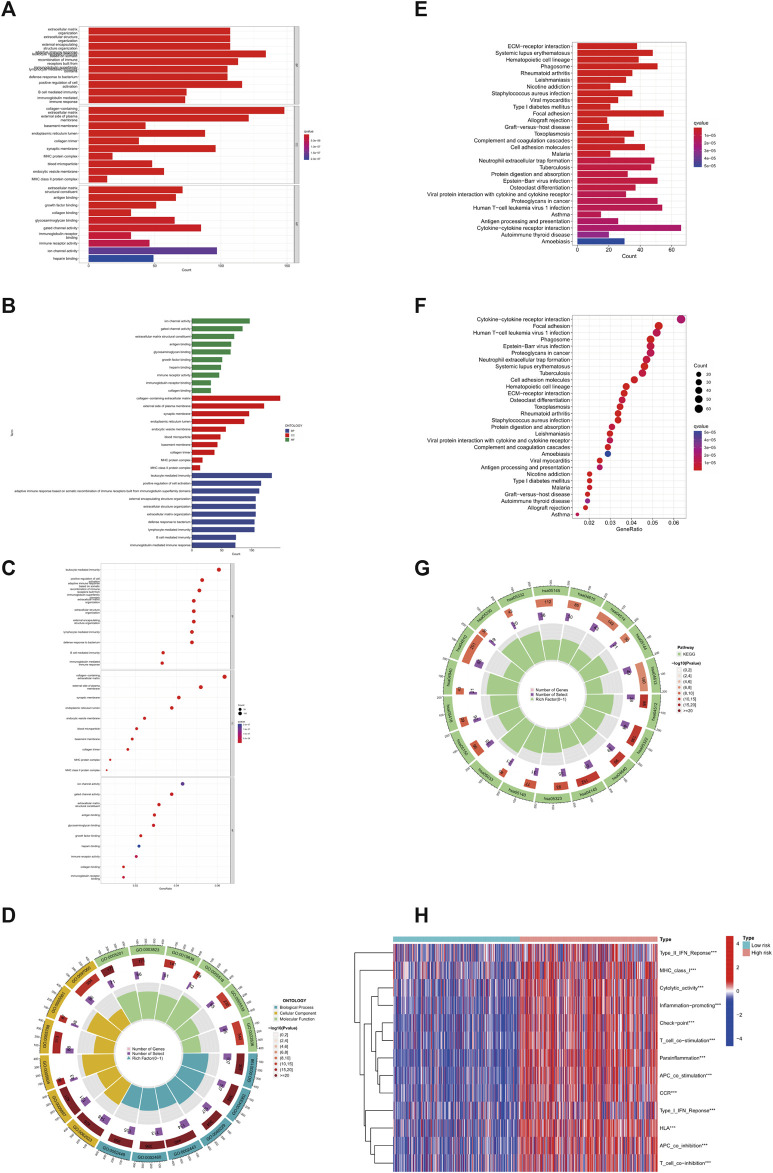
GLS involved in copper death of glioma cells. **(A–D)** GO enrichment analysis. **(E–G)** KEGG enrichment analysis. **(H)** Immune-related analysis. ***p < 0.001, **p < 0.01, *p < 0.05. Abbreviation: GO, Gene Ontology. KEGG, Kyoto Encyclopedia of Genes and Genomes.

To further explore the mechanism of GLS affecting glioma, we performed GO enrichment analysis, KEGG enrichment analysis, and immune-related functional analysis. In the GO analysis, the top 10 signalling pathways affected by GLS were mainly leukocyte mediated immunity, adaptive immune response based on somatic, B cell mediated immunity, and glycosaminoglycan binding, etc. (Figures 4A–D). With regard to the KEGG enrichment analysis, from the results obtained, we found that GLS was also significantly correlated with immunity and glucose metabolism (Figures 4E–G). In addition, we performed immune-related function analysis, and discovered that the differences in the levels of Type_II_IFN_Reponse, MHC_class_I, Parainflammation, etc. were highly significant (*p* < 0.001) in the high-risk group compared to the low-risk group of patients (Figure 4H). This suggests that GLS has a great relationship with the immune and glucose metabolic processes of glioma cells and is involved in mediating the cuproptosis process of glioma cells.

### GLS is involved in tumor mutation in glioma cells and affects the prognosis of glioma patients

**FIGURE 5:**
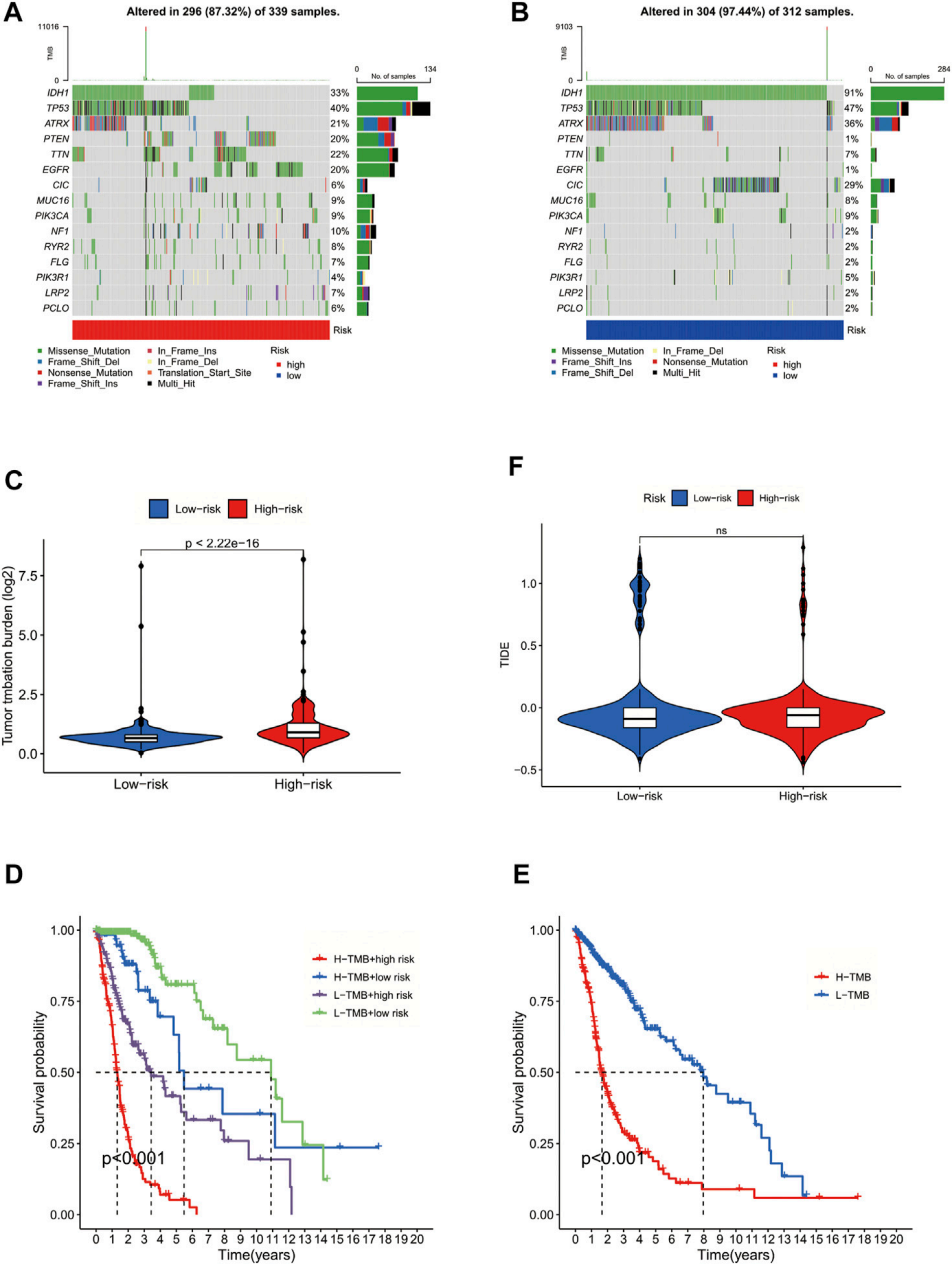
GLS involved in tumour mutation in glioma cells. **(A)** Forest plot of high-risk group. **(B)** Forest plot of low-risk group. **(C)** Violin plot of differential analysis of tumour mutation burden. **(D)** Survival curve of patients in high and low mutation load groups. **(E)** Survival curve of patients in high and low mutation load and high- and low-risk groups. **(F)** Violin plot of immune escape.

To further investigate the role of GLS in tumour mutation and immune escape in glioma cells, we obtained the mutational burden data of TCGA-GBM and TCGA-LGG and organised those data into a matrix of information available for analysis, with a total of 991 cases. We found that the frequency of gene mutations was generally higher in the high-risk group of glioma patients than in the low-risk group. However, there were exceptions; for example, IDH1 and TP53 were mutated at a higher rate in the low-risk group than they were in patients from the high-risk group (Figures 5A,B). In addition, we also performed a differential analysis of the tumour mutation load of patients in the high- and low-risk groups, finding that there was a significant difference in the glioma tumour mutation burden of patients in the high- and low-risk groups (*p* < 0.001), with patients in the high-risk group having a higher tumour mutation load than those in the low-risk group (Figure 5C). Moreover, the prognostic survival time of patients in the high mutation burden group was also significantly different from that in the low mutation burden group, with patients in the high mutation burden group having a significantly lower survival time than those in the low mutation burden group. These results suggest that the cuproptosis-associated gene GLS promotes tumour mutation in glioma cells and is detrimental to patients’ prognosis and survival (Figures 5D,E). Subsequently, immune escape and immunotherapy data for glioma were obtained from the TIDE database, and based on the results of the TIDE database scores, we found no statistical difference between the scores of patients in the high-risk and low-risk groups, indicating that there was no significant difference in the effectiveness of immunotherapy received by the two groups (Figure 5F). In summary, for patients in the high-risk group with high GLS expression, immunotherapy may still be a treatment option worth considering.

## Discussion

Currently, the main treatment for glioma is surgery, but the survival of patients after surgery remains poor (Ghouzlani et al., 2021) (Malta et al., 2018). Therefore, it is important to explore the specific mechanisms of glioma and to identify useful models to predict glioma prognosis. Intracellular copper accumulation triggers the aggregation of mitochondrial lipidylated proteins and destabilisation of iron-sulfur cluster proteins, leading to a distinct type of cell death called cuproptosis (Tang et al., 2022). Typically, elesclomol-mediated cancer cytotoxicity is associated with ferritin-1 (FDX1) levels and increased mitochondrial respiration rate, whilst it is also dependent on copper availability (Li et al., 2022) (Wang et al., 2022). The discovery of cuproptosis has provided new ideas for the treatment of glioma, and, indeed, all tumours. Therefore, we believe that further studies on cuproptosis are necessary. In the present work, we revealed that the GLS gene has substantial clinical effects and can be used as an independent predictor of survival outcome. Further analysis showed that the GLS gene is also involved in the cuproptosis process, tumour mutation, and immune escape in glioma cells.

First, GLS, a gene associated with cuproptosis, was found to be of key clinical relevance and could be used as an independent predictor of survival in glioma patients. Based on the analysis of glioma clinically-relevant cohort data from the TCGA database, we developed a cox model for the prognosis of patients with GLS gene-related glioma, and performed survival analysis and principal component analysis. Based on the principal component analysis, we distinguished cuproptosis-related lncRNAs into high- and low-risk groups and used this as a scoring basis. Subsequently, we performed a risk analysis based on the prognostic model and classified glioma patients into high- and low-risk groups according to the previous scoring and risk analysis results. We found that there was an interaction between GLS and high-risk cuproptosis-related lncRNAs, whilst the relationship between high and low GLS gene expression and patient prognosis was highly significant, and high expression of GLS genes promoted the cuproptosis process in glioma patient cells, which was detrimental to patient prognosis.

Another important finding of this study is that the GLS gene related to cuproptosis is also involved in tumour immunity and tumour mutation of glioma cells. It is mainly involved in T cell-mediated tumour immunity, Type_II_IFN_Response, MHC_class_I, Parainflammation and other immune processes. Additionally, we found that patients with high expression of GLS had a higher risk of immune escape and the worst efficacy of immunotherapy. Subsequently, through our further research and analysis, we found that GLS is also a high-risk factor for glioma mutation. The tumour mutation rate and the number of mutation genes in the GLS-related high-risk group were significantly higher than those in the low-risk group. The results of tumour mutation load survival analysis showed that the expression of GLS and tumour mutation load were significantly related to the prognosis. The survival time of patients in the high-risk and high mutation load group was the shortest and the prognosis was the worst. These results indicate that GLS plays an important role in glioma-associated immune response and tumour mutation, whilst also providing a new idea for the mechanism research and treatment of glioma.

The present study has its own limitations and flaws. First, the study was conducted mainly using bioinformatics methods; therefore, laboratory-based experiments are needed to support these findings. To compensate for the aforementioned shortcoming, this study used the TCGA and TIDE dual databases and performed rigorous data analysis, whilst a considerable amount of literature was consulted for validation, in an effort to make the findings reliable. Second, cuproptosis is a novel concept, and although significant progress has been made in this field, its specific mechanisms need to be further investigated. This study used bioinformatics methods to explore the validation and provide a strong reference for further basic experiments to follow.

GLS is indeed involved in the cuproptosis of glioma cells and affects the prognostic survival of glioma patients. The findings generated by the current study will provide important insights into the development of immunotherapy for glioma and even other cancers based on the molecular level.

## Conclusion

Through a comprehensive bioinformatics analysis, we found that GLS influences the development of glioma through the cuproptosis pathway. Using a cox prognostic model established by univariate cox analysis, multi-factor cox analysis, and lasso regression, based on the prognostic model with principal component analysis, we discovered that cuproptosis lncRNAs such as DARS-AS1, UBE1D3-AS1, and LYRM4-AS1 in glioma were significant high-risk lncRNAs; we used this finding to group patients into high- and low-risk groups. We discovered that the prognostic survival time and tumour mutation of patients in the high-risk group were significantly different from those for patients in the low-risk group, with patients in the high-risk group having shorter prognostic survival time, higher risk of tumour mutation. These findings are potentially valuable not only for advancing our current understanding of the role of GLS, but also for its translational use in the prognosis and immunotherapy of glioma patients.

## Data Availability

Publicly available datasets were analyzed in this study. This data can be found here: TCGA database (https://portal.gdc.cancer.gov) and TIDE database (http://tide.dfci.harvard.edu/login/).
